# Microspheres with 2D rGO/Alginate Matrix for Unusual Prolonged Release of Cefotaxime

**DOI:** 10.3390/nano13091527

**Published:** 2023-05-01

**Authors:** Islam Gomaa, Merna H. Emam, Ahmed R. Wassel, Kholoud Ashraf, Sara Hussan, Haitham Kalil, Mekki Bayachou, Medhat A. Ibrahim

**Affiliations:** 1 Nanotechnology Research Centre (NTRC), The British University in Egypt, El-Shorouk City, Suez Desert Road, Cairo 11837, Egypt; 2 Electron Microscope and Thin Film Department, Physics Research Division, National Research Centre, Dokki, Cairo 12622, Egypt; 3 Department of Biotechnology, Faculty of Agriculture, Ain Shams University, Cairo 11241, Egypt; 4 Biophysics Department, Mansoura University, Mansoura 35516, Egypt; 5 Chemistry Department, Faculty of Science, Suez Canal University, Ismailia 41522, Egypt; 6 Chemistry Department, Cleveland State University, Cleveland, OH 44115, USA; 7 Department of Inflammation & Immunity, Lerner Research Institute, Cleveland Clinic, Cleveland, OH 44195, USA; 8 Molecular Spectroscopy and Modeling Unit, Spectroscopy Department, National Research Centre, 33 El-Bohouth St., Dokki, Giza 12622, Egypt

**Keywords:** microspheres, alginate, cefotaxime, graphene oxide, controlled release

## Abstract

A synergistic interaction between reduced graphene oxide (rGO) and a biodegradable natural polymer, sodium alginate, was developed to create unique microspheres with protruding spiky features at the surface (spiky microspheres) that act as a super encapsulation and sustained release system for the highly effective antibiotic cefotaxime. Three forms of microspheres, namely alginate (Alg), alginate-cefotaxime (Alg-CTX), and alginate-cefotaxime-reduced graphene (Alg-CTX-rGO) composites, were prepared using calcium chloride as a cross-linking agent. The microspheres were characterized using field emission scanning electron microscopy (FESEM), Fourier-transform infrared (FT-IR) spectroscopy, and X-ray diffraction to investigate their pores, roughness, surface morphology, functional groups, phase formation, purity, and structural properties. The membrane diffusion method was employed to determine the release profile of Cefotaxime from the fabricated microspheres. The antibacterial activities of CTX solution, Alg microspheres, Alg-CTX microspheres, and Alg-CTX-rGO microspheres were investigated against gram-negative bacteria (*Escherichia coli*) using the agar diffusion method on Muller–Hinton agar. The prepared samples exhibited excellent results, suggesting their potential for enhanced antibiotic delivery. The results demonstrated the potential of the microsphere 2D rGO/alginate matrix for enhancing cefotaxime delivery with an unusual, prolonged release profile.

## 1. Introduction

Two-dimensional (2D) allotropic structures have been used in numerous biological applications, demonstrating internationally certified safety profiles. Recently, a wide range of oral and topical drug delivery systems have been developed that provide sustained-release dosing, maintaining steady plasma drug levels for prolonged periods while reducing side effects and overcoming limitations of conventional immediate-release dosage forms [1,2]. Graphene’s unmodified basal plane sites, with free surface electrons, are hydrophobic, enabling the loading of active pharmaceutical ingredients (API) and covalent modifications. Newly created nanoscale biocompatible composites offer significant potential applications in biology and medicine, including gene and small molecule transport for targeted therapies [3,4]. Cefotaxime (CTX) sodium is a third-generation, semisynthetic cephalosporin that shows a broad antibacterial spectrum against killer and resistant gram-positive and gram-negative bacteria as well as some anaerobic bacteria. Its bactericidal activity is due to its stability to beta-lactamase, inhibiting bacterial cell wall formation. CTX is effective against a variety of bacterial strains that cause septicemia, lower respiratory tract, urinary tract, skin, soft tissue, bone, joints, and central nervous system infections. Furthermore, CTX is commonly administered intravenously or intramuscularly in dosing form, not available as an oral, medical batch bandage for skin or topical uses [5,6,7]. In general, the efficacy of cephalosporin as an antibiotic group is well-known without oral or well-established sustained-release encapsulation form compared to other antibiotic groups [8]. In addition, commercial forms of Cefotaxime are currently not available as an oral formulation [8].

Drug loading into polymeric nanoparticles and their targeted therapy at infected sites have recently received great attention. They provide reduced toxicity and good pharmacokinetics of loaded drugs. Moreover, they can protect their cargo against in vivo and in vitro degradation [9,10,11]. Biodegradable polymers can manage and limit medication release over a certain period, with an initial burst action to circumvent infections. Alginate (Alg) is a linear anionic polysaccharide found in brown algae. It is made from two co-polymers, mannuronic and guluronic acids. The distribution of these units determines the polymer’s degree of polymerization, rigidity, and gel-forming ability [12,13,14]. Alginate microspheres can be fabricated using divalent cations such as Ca^2+^, which expands its biological applications. Despite the biocompatibility and biodegradability of alginate, it lacks the mechanical strength and cell interaction ability that is required for tissue engineering applications. Polymers can be affected due to changes in the surroundings, and as a result, the cargo drug can be delivered [15,16]. These alginate-containing aggregates seem to mirror the microcolony structures of biofilms as observed in vitro. The obvious studies support the view that co-administration of antibiotics with alginate lyase, which degrades the exopolysaccharide produced by mucoid strains of P aeruginosa, might benefit cystic fibrosis (CF) patients by increasing the efficacy of antibiotics in the respiratory tract [17,18,19,20]. When alginate lyase and gentamicin were administered together, the apparent elimination of mucoid bacteria from biofilms was achieved, whereas the mucoid bacteria in most control biofilms treated only with gentamicin persisted [21,22]. Therefore, alginates are thought to be an effective carrier for the encapsulation of antibacterial agents with good in vitro activity, low toxicity, favorable pharmacokinetics, and better patient outcomes. Due to their notable benefits of hydrophilicity, biocompatibility, mucoadhesive characteristics, bioavailability, ecologically benign properties, and cost-effectiveness, alginates have been extensively researched [23,24]. Alginate biopolymers are used to encapsulate anticancer medications to improve their bioavailability, sustained release, pharmacokinetics, and bio-clearance [25]. They are employed for building micro- and nanosystems for controlled and targeted drug delivery for cancer therapy [26]. Notably, these nanomaterials can be used in the treatment of malignancies and tumors using photothermal, photodynamic, and chemo-dynamic methods. Future research should focus on developing novel alginate-based systems with the advantages of non-invasiveness, patient compliance, and ease of drug administration for targeted cancer therapy [11,27,28]. As a result, crucial factors such as mucosal permeability, gastrointestinal tract environment stability, and medication solubility should be considered [29]. Additionally, there are still issues in optimizing synthesis procedures and conducting thorough clinical studies. Alginate gels are perfectly suited for the quick diffusion of loaded molecules because of their nano-porous structure [30,31]. A range of stimuli-sensitive nanosystems for medical therapy and diagnosis have been developed using alginate hydrogels as an ideal matrix for immobilizing proteins, responsive polymers, and nanomaterials [32,33,34]. To improve mechanical strength and cell contact, alginates are frequently mixed with carbon-based materials, bioglass, protein, ceramic, and other types of polymers. The incorporation of graphene in alginate-based systems offers the potential for enhanced drug delivery and release kinetics. The graphene calcium alginate–polymer blend sphere reported in this study is a novel delivery system designed to explore oral or topical delivery and release of Cefotaxime. This study paves the way for future therapies due to excellent flexibility, safety, cost, and green synthesis.

Here, we developed a novel synergistic interaction between rGO and sodium alginate to create spiky microspheres that serve as an advanced encapsulation and sustained release system for the potent antibiotic, Cefotaxime. Three microsphere formulations were prepared: Alg, Alg-CTX, and Alg-CTX-rGO composites, using calcium chloride as the cross-linking agent. The release profile and antibacterial activities of the three microsphere formulations were evaluated against gram-negative bacteria (*Escherichia coli*), demonstrating the potential of the Alg-CTX-rGO system for enhanced antibiotic delivery through a sustainable release mechanism.

## 2. Material and Methods

### 2.1. Materials

Sodium alginate (Alg) and calcium chloride dehydrate (CaCl_2_ ≥ 99%) were purchased from Fisher Scientific, Waltham, MA, USA. Cefotaxime sodium powder for injections (CTX, batch number 200526) was purchased from UP Pharma, Assuit, Egypt.

### 2.2. Synthesis of Graphene Oxide Sheets

GO was synthesized using the Hummers method [35,36]. Throughout this process, 1 g of graphite was first mixed with 35 mL H_2_SO_4_ and 3 g of KMnO_4_ and afterward stirred for about one hour in an ice-water path at a low temperature (<20 °C). Subsequently, 30% of H_2_O_2_ (105 mL) was carefully added to the above solution and stirred for an hour, then heated to approximately 100 °C. The reaction mixture was diluted by adding 280 mL of distilled water. Then it was filtered and kept overnight in a dryer at 70 °C. The GO was probe-sonicated for 20 min under pulsed mode (operating at an amplitude of 40%) (20 KHz), then sputtered in a wide glass dish in a vacuum oven overnight at 80 °C. It was later again probe-sonicated for 60 min under pulsed mode (operating at an amplitude of 40%) (20 KHz), then dried in a wide glass dish in a vacuum oven at 80 °C overnight to obtain reduced graphene oxide.

### 2.3. Preparation of Sodium Alginate Microspheres

Alg microspheres were prepared by dissolving 0.5 g Alg in 75 mL distilled water until complete dissolution. CaCl_2_ solution was prepared by dissolving 2 g CaCl_2_ in 150 mL distilled water. Alg solution was added dropwise using a 50 mL burette into a beaker containing CaCl_2_ solution. The obtained microspheres were then washed thoroughly several times with distilled water and air-dried.

### 2.4. Preparation of Alginate-Cefotaxime (Alg-CTX) Microspheres

CTX sodium solution was prepared by dissolving 0.5 g in 5 mL of distilled water. The prepared CTX solution was added into Alg solution while stirring and further followed the previous procedure as described in Section 2.3.

### 2.5. Preparation of Alginate-Cefotaxime-Reduced Graphene (Alg-CTX-rGO) Microspheres

rGO-Alg solution (20%, *w*/*v*) was prepared and then added dropwise into a beaker containing CaCl_2_ solution and further followed the previous procedure as described above to obtain the Alg-CTX-GO product.

### 2.6. In Vitro Release Study

The release profile of CTX from Alg-CTX and Alg-CTX-rGO microspheres was determined using the membrane diffusion method. A total of 25 mg of Alg-CTX and Alg-CTX-rGO microspheres were placed in a dialysis bag containing 10 mL of distilled water (12–14 kDa MWCO, Visking dialysis tubing, SERVA, Heidelberg, Germany). Then, the dialysis bag was inserted and fully immersed in 50 mL of distilled water as a release medium at 37 °C and pH 7. At various time intervals, 3 mL aliquots of the release medium were collected, and measured the absorbance using a spectrophotometer at a wavelength of 276 nm to determine the concentration of CTX.

### 2.7. Antibacterial Activity Study

Antibacterial activities of CTX solution, Alg microspheres, Alg-CTX microspheres, and Alg-CTX-rGO microspheres were tested against gram-negative bacteria (*Escherichia coli*) using the agar diffusion method on the Muller–Hinton agar [37]. An agar plate was uniformly swabbed with a bacterial solution using a sterilized cotton swab. The tested samples were placed on the prepared agar. A solution of CTX with a concentration of 1 mg/mL was prepared in Milli-Q water and loaded into a well (8 mm diameter) that was cut using a sterile cork borer on the agar. Prepared spheres containing equal amounts of CTX (1 mg CTX) of Alg, Alg-CTX, and Alg-CTX-rGO microspheres were placed on the agar. The plate was then incubated at 37 °C for 24 h. The dimensions of the inhibition zones around the samples were measured and recorded after incubation.

### 2.8. Characterization Techniques

The morphology and particle size of the prepared samples were characterized using field-emission scanning electron microscopy (FE-SEM, QUANTA FEG250, Thermo Scientific, Waltham, MA, USA). FTIR spectra were collected using an FTIR spectrometer the spectra were measured in the 4000 to 400 cm^−1^ wavenumber regions using a FTIR spectrometer (Vertex 70, Bruker, Germany); coupled with Platinum Diamond ATR, which consists of a diamond disc as an internal reflection element. The prepared sample was placed on the ATR crystal, and then the spectrum was recorded. The spectrum of air was used as a background before each sample analysis. Background and sample spectra were taken in a room with a temperature around 21–23 °C, at a spectral resolution of 4 cm^−1^, and for each measurement, 32 scans were performed. The X-ray diffraction (XRD) of the as-prepared microspheres was collected on a Malvern Panalytical Empyrean 3 diffractometer to determine the phase compositions and crystal structures. UV-Vis absorption spectra of the concentration change of the drug were measured using a double-beam spectrophotometer (Cary 5000 UV-Vis-NIR, Agilent Technologies, Santa Clara, CA, USA).

## 3. Results and Discussions

### 3.1. Preparation of Microspheres

The visual observation of the developed microspheres showed that they were hard and firm. The Alg microspheres had a yellow color that turned into yellowish orange upon the addition of CTX. The Alg-CTX-rGO microspheres appeared black. Observed fine structural features of the samples suggested that the addition of CTX and rGO were evenly dispersed in the alginate solution without any clump formation.

### 3.2. The Topographical Properties and Surface Roughness of Microspheres

**Figure 1:**
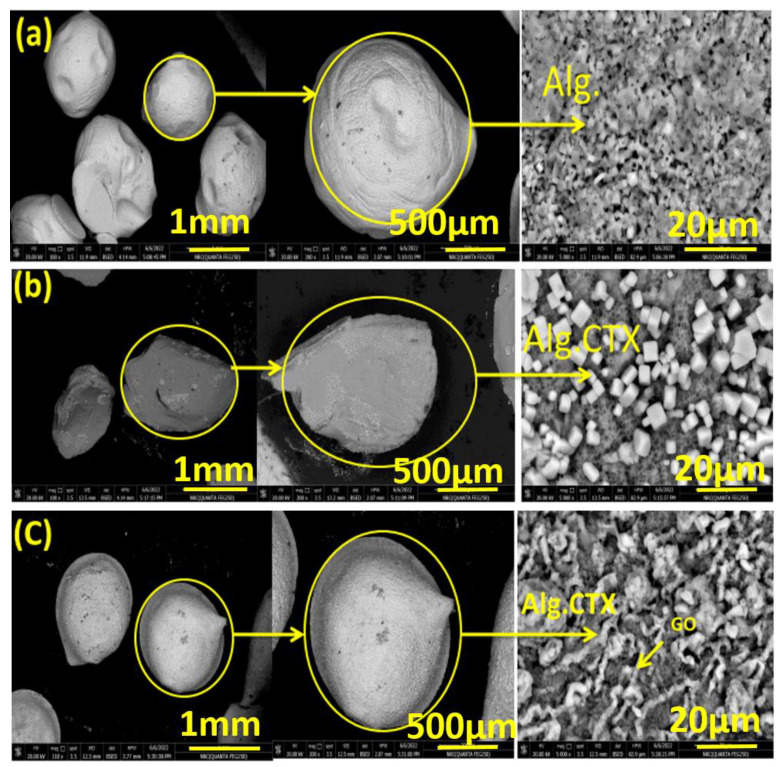
SEM micrographs of (**a**) Alg (**b**) Alg-CTX (**c**) Alg-CTX-rGO. All samples are as prepared using the procedures described in the experimental section.

**Figure 2:**
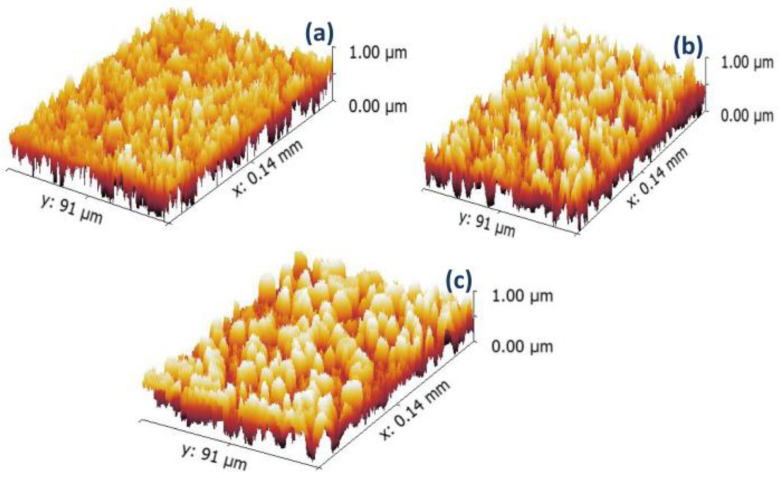
3D FE-SEM images of (**a**) Alg, (**b**) Alg-CTX, and (**c**) Alg-CTX-rGO. The 3D images are rendered and characterized using Gwyddion software.

The surface morphology and shape of the alginate microspheres were examined using FE-SEM, as shown in Figure 1. The surface of the alginate microspheres appears to be rough. The roughness increases with higher concentrations of drug and rGO in the alginate matrix. CTX drug crystals on the microsphere surface contributed to increased surface roughness. When the drug and rGO are added to sodium alginate, tighter and denser bonding is observed, resulting in a more homogeneous surface appearance. To better characterize the surface roughness of the microspheres, the 2D FE-SEM image was transformed into a 3D image using Gwyddion 2.5.1 software. This allowed for the assessment of roughness parameters such as roughness average (Ra), root mean square roughness amplitude (Rq), and maximal roughness valley depth (Rv), as shown in Figure 2 and Table 1. The results showed that increasing concentrations of the drug and rGO resulted in an increase in surface roughness, as indicated by the roughness parameter values. The sphere shape was influenced by the viscosity of the alginate and CaCl_2_ solution. Deformation increases as the viscosity of the gelling bath increases but could be compensated by increasing the viscosity of the alginate solution. Alginate flow rate and nozzle size have a stronger impact on the droplet diameter. Spherical beads were obtained if the droplets could cross the CaCl_2_ gelling bath surface and the impact with the liquid surface did not deform the droplet. The latter condition was improved, especially when the viscosity of the alginate solution was higher.

### 3.3. Fourier-Transforms Infrared Spectroscopy (FT-IR)

**Figure 3:**
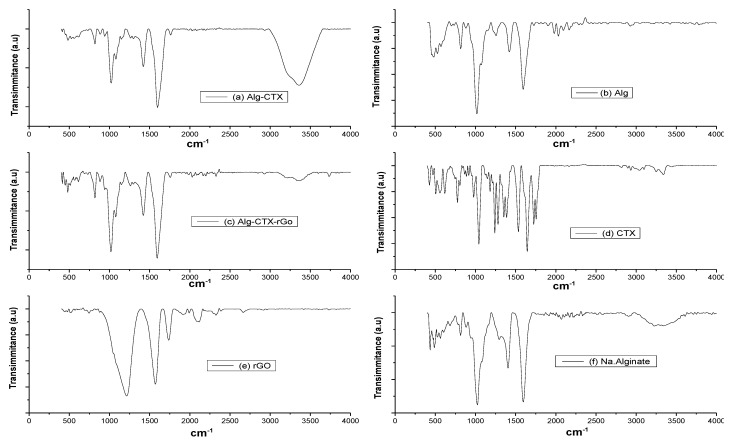
FT-IR spectra of (**a**) Alginate-Cefotaxime (Alg-CTX), (**b**) Alginate (Alg), (**c**) Aginate-Cefotaxime-rGO (Alg-CTX-rGO) microspheres and (**d**) Cefotaxime (CTX), (**e**) reduced graphene oxide (rGO) and (**f**) sodium alginate (Na.Alginate) powders Cef is the loaded drug Cefotaxime (CTX).

To investigate the chemical interaction of Alg, rGO, and CTX in microspheres, FTIR analysis was performed. The FTIR spectra of Alg, rGO, CTX, and Alg-rGO were similar to those reported in previous studies [38,39,40]. In Figure 3, the CTX spectra showed characteristic bands at 3389 and 1395 cm^−1^, attributed to O-H and C-H vibrational stretching, respectively. The absorptions at 1705, 1667, and 1621 cm^−1^ represent C–O conjugated to C–C, corresponding to GO. Additionally, the characteristic peaks at 1734 cm^−1^ and 1616 cm^−1^ are due to the C–O stretching of the carboxyl group and the C-C stretching of sp^2^ hybridized carbon atoms of embedded graphene. Peaks present at 1362, 1216, and 1044 cm^−1^ can be ascribed to carbonyl, epoxy, and alkoxy groups of rGO, respectively.

### 3.4. X-ray Diffraction (XRD) Spectroscopy

**Figure 4:**
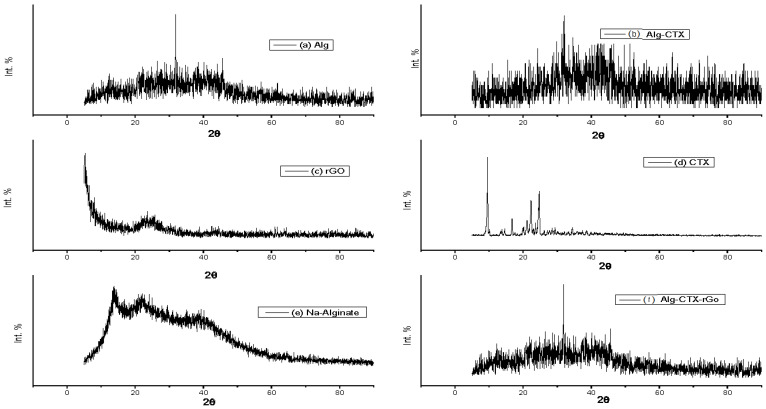
XRD patterns of (**a**) alginate sphere (Alg), Cefotaxime in (**b**) alginate matrix sphere (Alg-CTX-M), (**c**) reduced graphene oxide (rGO), (**d**) sodium Cefotaxime powder (CTX), (**e**) sodium alginate powder (Na.Alginate), and (**f**) Cefotaxim-reduced graphene oxide in alginate sphere matrix (Alg-CTX-g).

XRD analysis was performed to study the phase formation, purity, and structural properties of the prepared samples. As shown in Figure 4, the XRD pattern for cross-linked alginate revealed a semi-crystalline broad diffraction peak from 13° to 40°, along with a weak and broad shoulder peak at 32°. When compared to the sodium alginate spectrum, it suggests a decrease in amorphous structure hump and an increased crystallinity of the compound. These results are consistent with previous studies [41,42]. Figure 4 also shows the XRD pattern of rGO, which exhibits a broad characteristic peak at a 2θ value of 22.5–25°, suggesting graphene reduction. This peak could be due to the interaction of oxygen groups present in the graphene layers. A sharp peak at 26.8° is also observed, which might be due to the unreacted graphite. However, the addition of Cefotaxime significantly affected the Ca^2+^ cross-linked Alg-rGO microspheres, as indicated by similar broad peaks signifying the low crystallinity of the material.

### 3.5. In Vitro Drug Release Study

**Figure 5:**
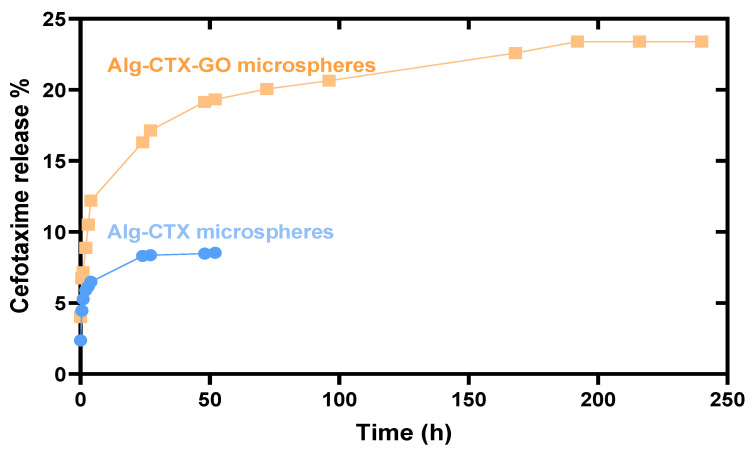
Release profiles of CTX from Alg-CTX, and Alg-CTX-rGO microspheres.

Both formulations exhibited a slow and sustained release of CTX, Figure 5. The incorporation of rGO into the microspheres resulted in a prolonged release over approximately 10 days (240 h), with 23% of CTX released from Alg-CTX-GO microspheres versus approximately 8.5% of CTX released over 52 h from Alg-CTX microspheres.

The results demonstrate a sustained release behavior, with about 16.3% of CTX released from Alg-CTX-rGO microspheres after 24 h. This is to be compared with just about half the amount of total CTX released (8.3%) from Alg-CTX microspheres over the same duration, Figure 5. Both formulations showed a slow and sustained release of CTX. This behavior could be due to the dispersion of the rGO nanosheets in the microsphere structure, as suggested by the surface analysis in Figure 1, which shows an increase in free spaces and pores within the microspheres, which is expected to lead to a larger loading of cefotaxime crystals within microspheres. Furthermore, the addition of rGO nanosheets causes enhancement in the number of the hydrophilic functional groups (COOH and OH), which enhances the interaction with the cefotaxime molecules as a drug cargo within the rGO-modified alginate microspheres.

The sustained release behavior is a positive indication, suggesting the suitability of the modified alginate system as a drug delivery vehicle for the treatment of resistant types of bacterial skin infections, particularly for people who suffer from immunodeficiency and require prolonged antibiotic treatment time. In comparison to other works outlined in Table 2, our rGO-Alg matrix shows an approximately tenfold increase in release time compared to previously reported systems.

### 3.6. Antibacterial Activity Study

**Figure 6:**
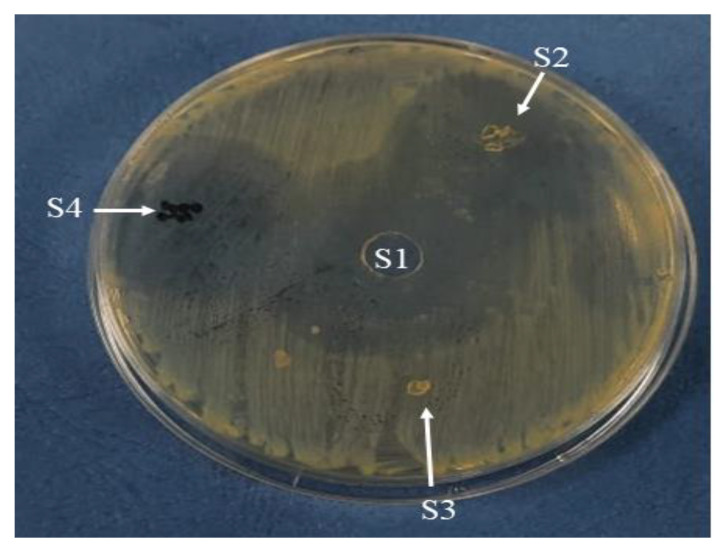
Antimicrobial activity test by agar diffusion method in *E. coli* culture. S1–S4 are inhibition zones developed after treatments with S1 through S4 as indicated in Table 3.

The antibacterial activities of CTX solution, Alg, Alg-CTX, and Alg-CTX-rGO microspheres were evaluated against *E. coli* by examining the presence or absence of inhibition zones. The findings presented in Table 3 highlight the promising antibacterial activity of Alg-CTX and Alg-CTX-rGO microspheres against *E. coli*, as evidenced by their 3 cm diameter zones of inhibition. Figure 6 further illustrates the differences in growth inhibition zones achieved by the tested samples, demonstrating the efficacy of these novel microsphere formulations. This observation is likely a result of the sustained release of CTX from the Alg-CTX and Alg-CTX-rGO microspheres. These results are consistent with the work of Tu et al., who reported pristine graphene nanosheets cause the degradation of both the outer and inner membranes of *E. coli* [49]. Similarly, Aunkor et al. demonstrated that rGO exhibited strong antimicrobial activity against the gram-positive and gram-negative multi-drug drug-resistant (MDR) bacteria [50]. Liu et al. reported that rGO oxidizes glutathione, which serves as a crucial redox mediator in bacteria, leading to bacterial death [51]. They suggested that the possible antimicrobial mechanism of rGO is membrane and oxidation stress. Combining CTX with rGO, which has a dual impact, is likely to inhibit the growth of bacterial strains which feature resistance characteristics. The efficiency of this system is likely due to the different intrinsic mechanisms of antimicrobial activity of its components as well as an appropriately distributed concentration release. Furthermore, loading them into spiky microspheres offers the advantage of providing sustained antimicrobial action.

## 4. Conclusions

Alginate spheres were successfully tuned as drug delivery release systems by incorporating rGO using a green and simple method to form unique microspheres with protruding spiky features on the surface (spiky microspheres) with superior encapsulation properties for the highly effective and remarkable antibiotic cefotaxime. We developed two forms of microspheres and compared their characteristics: Alg-CTX and Alg-CTX-rGO composites. FESEM revealed that drug crystals appeared on the surface of microspheres, leading to an increase in surface roughness. The presence of rGO and the drug in the sodium alginate solution resulted in tighter and denser bonding with a more homogeneous surface. FT-IR analysis established the combination of the individual components (Alg, CTX, and rGO) through the generation of new vibrations of CTX and rGO functional groups at 3389 and 1395 cm^−1^ (due to O-H and C-F vibrational stretching, respectively), and the absorption at 1705, 1667, and 1621 cm^−1^ (representing C–O conjugated to C–C, respectively). The membrane diffusion method was employed to determine the release profile of CTX from the fabricated microspheres, which showed a release time of around 240 h, which is at least ten times longer than other known releases by previously reported systems. Antibacterial activities against gram-negative bacteria (*E. coli*) using the agar diffusion method showed that Alg-CTX and Alg-CTX-rGO microspheres exhibit very promising antibacterial activity against *E. coli*.

## Figures and Tables

**Table 1:** The estimated roughness parameters of the prepared samples (a) Alg (b) Alg-CTX (c) Alg-CTX-rGO.

Sample	Roughness Parameter		
	Average Roughness R_a_ (nm)	Root Mean Square Roughness R_q_ (nm)	Maximum Roughness Valley Depth R_v_ (nm)
Alg.	4.62	6.2	2.09
Alg.-CTX.	4.69	6.23	4.46
Alg.CTX.rGO	4.92	6.23	4.85

**Table 2:** Comparison of release time of CTX from the rGO-Alg system and previously reported systems.

Drug	Method	Release Time (Up to)	Reference
Cefotaxime	Zn-Al Layered double hydroxides (LDH) with polyvinyl alcohol (PVA)	12 h	[43]
Cefotaxime	Mg-Al LDH with fenugreek polymer (CLF nanohybrid)	18 h	[44]
Cefotaxime	Polycaprolactone (PCL) nanoparticles	48 h	[45]
Cefotaxime	High-molecular-weight polylactide (PLA) films	24 h	[46]
Cefotaxime	MNPs by poly (N, Ndimethylaminopropylacrylamide)(pDMAPAAm)	15 h	[47]
Cefixime	Ethyl cellulose microspheres	24 h	[48]
Cefotaxime	Alginate microspheres	52 h	this work
Cefotaxime	Alginate-graphene microspheres	240 h	this work

**Table 3:** Growth inhibition zones (cm) of CTX solution, Alg, Alg-CTX, and Alg-CTX-rGO microspheres against *E. coli*.

Sample	*E. coli*
S1	4 cm
S2	3 cm
S3	-ve
S4	3 cm

S1: CTX solution, S2: Alg-CTX-rGO microspheres, S3: Alg microspheres, and S4: Alg-CTX microspheres.

## Data Availability

Data of research in this work is available upon request.
